# Body Weight’s Role in Infective Endocarditis Surgery

**DOI:** 10.3390/jcdd11100327

**Published:** 2024-10-15

**Authors:** Ahmed Elderia, Gerold Woll, Anna-Maria Wallau, Walid Bennour, Stephen Gerfer, Ilija Djordjevic, Thorsten Wahlers, Carolyn Weber

**Affiliations:** Department of Cardiac Surgery, Heart Center, University of Cologne, Kerpener Straße 62, 50937 Cologne, Germany

**Keywords:** infective endocarditis, valve surgery, BMI and body weight

## Abstract

**Objective:** to investigate how body mass index (BMI) affects the outcome in patients treated surgically for infective endocarditis (IE). **Methods:** This is a single-center observational analysis of consecutive patients treated surgically for IE. We divided the cohort into six groups, according to the WHO classification of BMI, and performed subsequent outcome analysis. **Results:** The patient population consisted of 17 (2.6%) underweight, 249 (38.3%) normal weight, 252 (38.8%) overweight, 83 (12.8%) class I obese, 28 (4.3%) class II obese, and 21 (3.8%) class III, or morbidly obese, patients. The median age of the entire cohort was 64.5 [52.5–73.6] years. While only 168 (25.9%) patients were female, women significantly more often exhibited extremes in regards to BMI, including underweight (47.1%) and morbid obesity (52.4%), *p* = 0.026. Class II and III obese patients displayed more postoperative acute kidney injury (47.9%), *p* = 0.003, more sternal wound infection (12.9%), *p* < 0.001, worse 30-day survival (20.4%), *p* = 0.031, and worse long-term survival, *p* = 0.026, compared to the results for the other groups. However, the multivariable analysis did not identify obesity as an independent risk factor for 30-day mortality, with an odds ratio of 1.257 [0.613–2.579], *p* = 0.533. Rather, age > 60, reduced LVEF < 30%, staphylococcal infection, and prosthetic valve endocarditis correlated with mortality. While BMI showed poor discrimination in predicting 30-day mortality on the ROC curve (AUC = 0.609), it showed a fair degree of discrimination in predicting sternal wound infection (AUC = 0.723). **Conclusions:** Obesity was associated with increased comorbidities, complications, and higher postoperative mortality in IE patients, but it is not an independent mortality risk factor. While BMI is a poor predictor of death, it is a good predictor of sternal wound infections.

## 1. Introduction

Infective endocarditis (IE) is a life-threatening disease, usually presenting with a severe course and a wide range of complications [1]. Untreated, IE patients usually have a poor prognosis. Even with early and optimal therapy, the in-hospital survival rate is just over 80% [2,3]. Therefore, the identification of factors influencing the clinical course is of immense importance for clinical practice. Along with other clinical parameters, the body mass index (BMI) contributes to the overall risk stratification of a patient [4]. BMI is a simple and widely accepted anthropometric measurement of a patient’s body proportions, nutritional status, and overall physique [5]. Obesity is often associated with a variety of cardiovascular and endocrine comorbidities [6]. On the other hand, underweight patients might exhibit underlying malnutrition, immunosuppression, or sepsis and are susceptible to reduced wound healing and delayed recovery after surgery. Thus, it could be assumed that patients exhibiting extreme BMIs are at increased risk of a worse postoperative course after surgery for IE.

The impact of BMI on the outcomes of surgically treated patients with IE is not yet fully understood, as conflicting results exist in the literature. Harris et al. have found that higher BMI is associated with increased rates of postoperative complications after cardiac surgery, prolonged hospital stays, and higher resource utilization [7]. Conversely, Johnson et al. have shown no significant association between BMI and surgical outcomes, with even better survival after cardiac surgery, reporting a so-called “obesity paradox” [8]

The main objective of our analysis is to enhance the understanding of the characteristics and factors that influence the clinical course and the surgical outcomes of IE patients, specifically exploring the impact of BMI on postoperative outcomes. Therefore, we analysed (i) whether the patient population, subdivided by preoperative BMI, showed significant differences in terms of clinical presentation and comorbidities or (ii) whether they showed differences in short and long-term survival and (iii) whether BMI itself or other predictors influenced survival.

## 2. Patients and Methods

This is a single-centre observational analysis of consecutive patients treated surgically for IE since 2009. The local institutional ethics committees approved the study protocol on the 7 February 2018.

Infective endocarditis was defined according to the recently modified Duke criteria, and surgery was indicated in compliance with the current European Society of Cardiology guidelines [2,9]. All operations were performed under general anesthesia via median sternotomy, with routine establishment of cardiopulmonary bypass (CPB) techniques utilizing roller head pumps, a membrane oxygenator, cardiotomy suction, moderate systemic hypothermia (34 °C), and cardiologic arrest. The age of the patients was recorded at the time of surgery due to IE. The BMI was defined as the body mass divided by the square of the body height, obtained preoperatively, and expressed in kg/m^2^. The WHO classification of BMI guided our analysis, and is comprised six groups, as follows: [10].

Underweight: BMI < 18.5;

Normal weight: BMI ≥ 18.5–24.9;

Overweight: BMI 25–29.9;

Obesity class I: BMI 30–34.9;

Obesity class II: BMI 35–39.9;

Obesity class III: BMI ≥ 40 (morbid obesity).

Sepsis was defined as an acute onset of at least one or more organ dysfunction(s) in relation to infection. Shock was defined as hypotension with the need for vasopressors, despite adequate fluid resuscitation. Known previous cerebrovascular events (CVE) were defined as ischemic or hemorrhagic cerebral insults in relation to IE. Postoperative CVE was considered as any new-onset neurologic deficit of cerebral origin, in association with signs of hemorrhage or ischemia on a CT/MRI of the brain, along with an assessment by a neurologist that occurred during the primary hospital stay. Sternal wound infection was defined as the presence of clinical and microbiological signs of infection of the soft tissue and/or the sternum, ribs, costal cartilage, and/or mediastinum, occurring as a result of the influence of local or systemic factors subsequent to a median sternal incision, requiring surgical management. Acute renal failure was defined according to the Kidney International Supplements 2012 [11]. The 30-day and 1-year mortality rates included death from any cause within the first 30 days and between day 31 and day 365 after surgery, respectively. Late mortality was defined as all-cause mortality occurring during the follow-up period. The follow-up time for survival was measured from the date of the operation to either the date of death or the date of the last contact with the patient. Long-term follow-up was obtained by reviewing the hospital medical records and interviewing the patients, their relatives, or patients’ physicians. The median duration of the follow-up was 2.4 years [interquartile range (IQR) 0.26–5.8], with a completeness of 73.6%.

### Statistical Analysis

All data were statistically analyzed using SPSS^®^ Statistics version 28.0 (IBM Corporation, Armonk, NY, USA). The Shapiro–Wilk test was used to assess normal distribution. Depending on the distribution, continuous variables were expressed as the mean and standard deviation or the median with the respective interquartile range. Group comparisons were performed using the one-way ANOVA or Kruskal–Wallis ANOVA test. Discrete variables were expressed as percentages and were tested using the Chi-squared or Fisher’s exact test. Missing data were not imputed and were randomly assumed to be missing. Potential risk factors for 30-day mortality (days 1 to 30) were assessed using logistic regression, and potential risk factors for 1-year mortality (days 31 to 365) were evaluated using Cox regression. After univariable analysis, all variables with a *p* value less than 0.1 were entered into the multivariable model using a forward selection (likelihood ratio of *p* less than 0.05). The results are presented as the odds ratio (OR) for 30-day mortality or the hazard ratio (HR) for 1-year mortality, with the corresponding 95% confidence interval (CI) and *p* value. All reported *p* values are two-sided and are considered statistically significant at 5% or less. In addition, a Kaplan–Meier analysis was performed to examine long-term survival. To better understand the association between BMI and survival, we also performed a regression scatterplot. The predictive value of BMI was tested by constructing a receiver operating characteristics (ROC) curve. An area under the curve (AUC) of 0.7 or less was considered to show poor discriminatory power.

## 3. Results

The entire cohort comprised 650 patients who were surgically treated for IE at our institution between January 2009 and December 2019. The examined population included 17 (2.6%) underweight patients, 249 (38.3%) normal weight patients, 252 (38.8%) overweight patients, 83 (12.8%) class I obese patients, 28 (4.3%) class II obese patients, and 21 (3.8%) morbidly obese patients. The median age of the entire population was 64.5 [52.5–73.6] years, without statistical difference between groups. Although only 168 patients (25.9% of the total) were female, women were significantly more likely to display extreme BMI values, including underweight (47.1%) and morbid obesity (52.4%), *p* = 0.026. The mean EuroSCORE II in the entire population was 8.3 ± 3.7, without statistical difference between groups, with *p* = 0.197. Normal and underweight patients less frequently exhibited diabetes mellitus, arterial hypertension, and chronic kidney disease (CKD) than did obese patients. Class II and III obese patients, e.g., 39.3% and 47.6%, *p* = 0.047, respectively, more often exhibited hyperlipidemia (Table 1). The six groups were predominantly balanced concerning predisposing factors for IE (Supplementary Table S1). Mitral valve prolapse was more frequently noticed in the underweight group (29.4%, *p* < 0.001). Preoperatively, the highest prevalence of shock was observed in all groups among patients with morbid obesity (33.3%), *p* < 0.006. A further list of clinical manifestations of IE can be found in (Table 2). Echocardiographic and microbiological findings were not divergent between groups (Supplementary Table S2).

In the entire cohort, aortic valve IE (58.9%), followed by mitral valve IE (45.5%) were more common than right heart endocarditis (6.8%). Only in the underweight group, more patients exhibited mitral than aortic valve involvement (70.6% and 35.3%, respectively). Double-valve endocarditis was noted in 8.3% of patients (Table 3).

Half of the class III obese patients developed acute kidney injury (AKI) (52.4%), *p* = 0.011, while 21.4% acquired sternal wound infections, *p* = 0.002. In the whole cohort, the mean ICU stay was 5.0 ± 6.3 days, and the median in-hospital stay was 13 days [9.7–19.0], without significant differences between groups. Likewise, no differences were found in terms of readmission or the recurrence of IE (Table 4).

**Figure 1:**
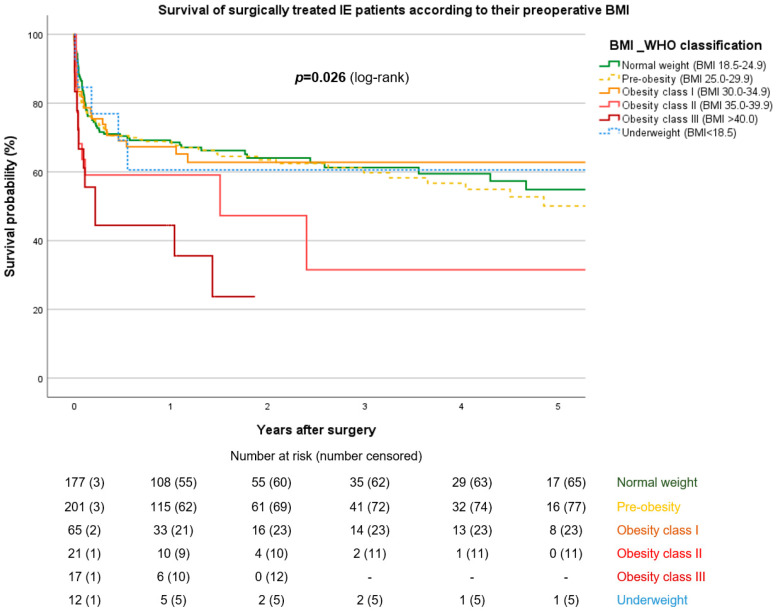
Kaplan–Meier survival estimation, according to BMI classes.

**Figure 2:**
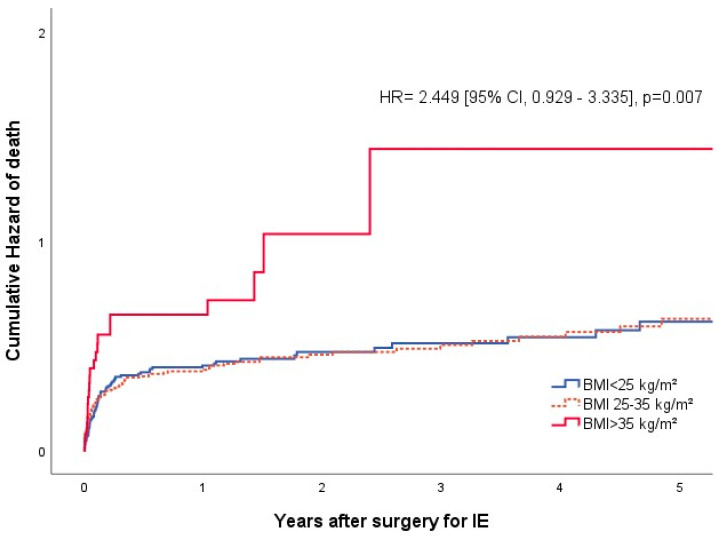
Survival estimation for the combined groups.

In the entire population, the 30-day mortality was 13.1%. Although not statistically significant, a nearly thrice as high 30-day mortality rate was observed in the obese II group (36.4%, *p* = 0.225). Combining the results for the class II and III obese patients and comparing them to the those for the remaining population showed that these patients exhibited a higher 30-day mortality rate of 20.4%, *p* = 0.031, OR 2.38 [1.19–4.74] (Supplementary Table S3). A Kaplan–Meier analysis revealed that patients with class II and III obesity expereinced significantly worse long-term survival (*p* = 0.026) (Figure 1). A significantly higher mortality hazard for class II + III patients was also noticed in the pooled subanalysis, with HR = 2.449 [0.929–3.335], *p* = 0.007 (Figure 2). The median survival in the class III group was 57 [15.0–228.5] days. Since less than 50% of patients died within the follow-up period in all other groups, no median survival rate can be obtained for these groups. Among all causes of death, sepsis and multi-organ failure were the most frequent causes, without differences between groups.

**Figure 3:**
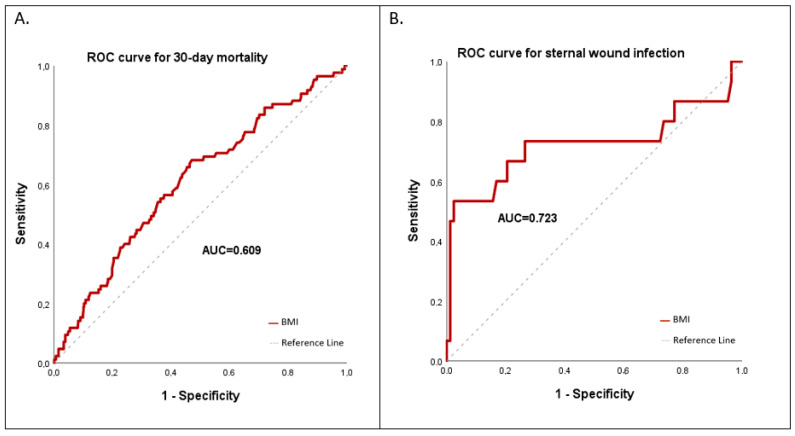
ROC curves for 30-day mortality (**A**) and for sternal wound infection (**B**).

Our multivariable analysis showed that abnormal BMI is not an independent risk factor for mortality (Table 5). Instead, our multivariable analysis showed that the following variables were independent predictors of increased 30-day or 1-year mortality: age over 60 years, infection with Staphylococcus aureus, reduced left ventricular ejection fraction (LVEF) < 30%, as well as prosthetic valve endocarditis (PVE) (Table 6). Moreover, a week correlation between BMI and survival time was visualized in a scatter plot (r^2^ = 0.01) (Supplementary Figure S1). While BMI showed poor discrimination ability in predicting 30-day mortality on the ROC curve (AUC = 0.609), it showed a fair degree of discrimination in predicting sternal wound infection (AUC = 0.723) (Figure 3). For the cut-off point BMI = 35 kg/m^2^, a sensitivity (true positive rate) of 53% and a 1-specificity (false positive rate) of 3.6% were calculated. The positive likelihood ratio was 14.7, while the negative likelihood ratio was 0.45.

## 4. Discussion

We present a comprehensive analysis on the association between BMI and the clinical presentation and postoperative course of IE patients. Our main significant findings included the following: (i) Although more males underwent surgery for IE, significantly more females in the study were either underweight or morbidly obese. Class II and III obese patients exhibited more comorbidities, as well as a more pronounced clinical course, with a higher rate of septic shock. (ii) Class II and III obese patients displayed worse long-term survival rates than did the other groups. (iii) Abnormal BMI could not be identified as an independent risk factor for mortality; however, it showed a fair degree of discrimination in predicting sternal wound infection.

Worldwide, body weight disorders are considered major public health problems. According to self-reported data from 2020, more than half of adults (53.5%) in Germany are overweight, and nearly one-fifth (18.1%) are obese (defined by WHO classification) [12]. Underweight or malnutrition disorders are much less prevalent. Compared to older data from 2012, the prevalence of obesity has continued to increase, particularly among 45- to 64-year-olds, and it is higher among men than women (60.5% vs. 46.6%) [13]. In contrast to the pattern regarding gender and body weight in the German population, more females exhibited morbid obesity in our examined cohort. In the underweight group, females and males were found to contribute equally. In the context of IE, many studies, like ours, showed that more men than women undergo surgery due to IE [14,15]. Supposedly, women with extreme BMIs are more susceptible to IE. Our data, however, do not allow for a definite prediction, and further studies are warranted.

Obese patients more often presented with diabetes mellitus, arterial hypertension, hyperlipidemia, and CKD. However, this multimorbidity did not lead to a difference in the calculated EuroSCORE II between the groups. Morbidly obese patients exhibited significantly higher instances of preoperative shock and were more often intubated and ventilated. Obesity is presumed to be associated with an inflammatory condition in which elevated levels of various pro-inflammatory cytokines and acute-phase proteins exacerbate oxidative stress and endothelial cell dysfunction [16]. This would explain why obese patients present with more signs and complications of sepsis, yet the impact on survival is controversial. A recent meta-analysis examined the association of morbid obesity and mortality in patients with sepsis and showed a non-significant association [17].

Class II and III obese patients exhibited higher rates of postoperative wound infection, acute kidney injury, and 1-year mortality. The ROC curve showed a good discrimination for BMI in predicting sternal wound infection. Higher wound infection rates in obese patients vs. non-obese patients are evidenced, as reported after cardiac surgery in many studies [18,19]. In agreement with our results, O’Sullivan reported higher rates of AKI after cardiac surgery in obese patients with a BMI ≥ 30 kg/m^2^ [20].

Regarding the impact of obesity on mortality after cardiac surgery, the evidence is controversial. Prabhakar et al. reported that morbid obesity seems to be an independent risk factor for mortality after coronary artery bypass grafting (CABG) surgery [21]. Other authors reported rather neutral results [22,23]. In an aortic valve surgery cohort, better long-term results postoperatively were shown in overweight and class I obese patients than in underweight and normal weight patients. The authors then commented on an “obesity paradox”, referring to better outcomes associated with higher BMI in patients undergoing cardiac surgery [24]. However, there are concerns that the observed decrease in mortality might be attributed to the research methodology used rather than to the actual increase in BMI [25].

On the other hand, the malnutrition and frailty that underweight patients may suffer is relevant perioperatively. Potapov et al., in their retrospective study of over 20,000 CABG patients, found that underweight patients had a higher risk of postoperative complications than did normal weight or even morbidly obese patients [19].

In the context of IE, a cohort study (n = 414) examined the predictors of long-term mortality in left-sided IE and found that higher BMI was an independent risk factor for long-term mortality in 1-year survivors [26]. Gatti et al., in a multicenter analysis, found that a BMI greater than 27 kg/m^2^ is an independent risk factor for in-hospital mortality. BMI was integrated into the five-parameter AEPEI score for evaluation of the perioperative mortality risk in IE patients [27]. In our analysis, BMI showed poor discrimination in predicting 30-day mortality, and abnormal BMI was not found to be an independent risk factor for mortality in the multivariable regression analysis. Rather, age over 60 years, IE caused by Staphylococcus aureus, LVEF < 30%, as well as PVE, were identified as independent risk factors for 30-day and 1-year mortality. All four variables are evidenced risk factor for higher mortality after surgery for IE [28,29,30,31]. The worse outcomes in obese patients may have been influenced by these variables. Both Staphylococcus aureus infection and LVEF < 30% were indeed more prevalent, in terms of percentages, in the obese II and III groups, but did not reach the significance level in the descriptive analysis.

## 5. Limitations

Our analysis includes some inherent limitations. The collected data originated from an unselected patient series from a single center. Although we present a relatively large cohort of surgically treated IE patients, when compared to the methods used in the available literature, the power of our analysis could have been increased by including a larger number of patients and conducting a multicenter study. The underweight and obesity class III groups included only 17 and 21 patients, respectively. This limited sample size could potentially have led to spurious negative results, indicating a risk of type II error. Therefore, in the subanalysis, we combined the groups to minimize this risk.

## 6. Conclusions

Obese patients with a BMI ≥ 35 kg/m^2^ exhibit more comorbidities and worse survival after surgery for IE. However, independent risk factors for increased mortality include older age, reduced LVEF < 30%, staphylococcal infection, and prosthetic valve endocarditis, rather than an abnormal BMI. While BMI is a poor predictor of mortality, it may be a fair predictor of sternal wound infection.

## Figures and Tables

**Table 1:** Baseline data of surgically treated IE patients, according to their BMI.

Variables	All Patients n = 650	Under-Weight 17/650 (2.6%)	Normal Weight 249/650 (38.3%)	Overweight 252/650 (38.8%)	Obese I 83/650 (12.8%)	Obese II 28/650 (4.3%)	Obese III 21/650 (3.2%)	*p*-Value
Age	64.5 [52.5–73.6]	63.0 ± 13.4	62.9 [47.8–73.1]	66.4 [43.3–74.8]	70.0 ± 10.5	61.1 ± 12.8	57.7 ± 8.2	0.082
Female gender	168/649 (25.9%)	8/17 (47.1%)	58/249 (23.3%)	60/252 (23.8%)	23/83 (27.7%)	8/27 (29.6%)	11/21 (52.4%)	**0.020**
LVEF < 30%	17/650 (2.6%)	0/17	4/249 (1.6%)	8/252 (3.2%)	2/83 (2.4%)	1/28 (3.6%)	2/21 (9.5%)	0.592
EuroSCORE II	8.3 ± 3.7	8.3 ± 2.9	7.9 ± 3.8	8.8 ± 3.7	8.3 ± 3.9	8.1 ± 3.4	8.0 ± 2.3	0.197
Aortic valve IE	392/650 (60.3%)	6/17 (35.3%)	142/249 (47.0%)	161/252 (63.9%)	56/83 (67.5%)	14/28 (50.0%)	13/21 (61.9%)	0.076
Mitral valve IE	303/650 (46.6%)	12/17 (70.6%)	114/249 (45.8%)	112/252 (44.4%)	38/83 (45.8%)	15/28 (53.6%)	12/21 (57.1%)	0.309
Tricuspid valve IE	42/650 (6.5%)	0/17	22/249 (8.8%)	13/252 (5.2%)	5/83 (6.0%)	1/28 (3.6%)	1/21 (4.8%)	0.451
Pulmonary valve IE	3/650 (0.5%)	0/17	2/249 (0.8%)	1/252 (0.4%)	0/83	0/28	0/21	1.000 *
Preoperative WBC (/µL)	10.2 ± 6.8	6.3 ± 2.7	9.9 ± 4.8	10.6 ± 8.9	9.5 ± 3.9	11.0 ± 6.0	11.9 ± 7.1	0.425
C-reactive protein (mg/L)	42.5 [9.5–120.7]	44.0 [8.5–137.0]	49.5 [9.4–156.0]	22.0 [5.7–141.0]	50.5 [8.5–116.7]	50.5 [9.2–112.5]	57.0 [28.0–137.0]	0.202
Diabetes mellitus	164/650 (25.2%)	0/17	39/249 (15.7%)	66/252 (26.2%)	36/83 (43.4%)	15/28 (53.6%)	8/21 (38.1%)	**<0.001**
Arterial hypertension	410/650 (63.1%)	7/17 (41.2%)	124/249 (49.8%)	175/252 (69.4%)	65/83 (78.3%)	21/28 (75.0%)	18/21 (85.7%)	**<0.001**
Hyperlipidaemia	185/650 (28.4%)	4/17 (23.5%)	58/249 (23.3%)	73/252 (29.0%)	29/83 (34.9%)	11/28 (39.3%)	10/21 (47.6%)	**0.047**
CAD	187/650 (28.7%)	2/17 (11.8%)	70/249 (28.1%)	77/252 (30.6%)	23/83 (27.7%)	8/28 (28.6%)	7/21 (33.3%)	0.684
Atrial fibrillation	28/650 (4.3%)	1/17 (5.9%)	11/249 (4.4%)	7/252 (2.8%)	3/83 (3.6%)	3/28 (10.7%)	3/21 (14.3%)	0.091
Pulmonary hypertension	50/650 (7.7%)	0/17	23/249 (9.2%)	19/252 (7.5%)	6/83 (7.2%)	1/28 (3.6%)	1/21 (4.8%)	0.668
Cerebrovascular disease	84/650 (12.9%)	4/17 (23.5%)	25/249 (10.0%)	37/252 (14.7%)	14/83 (16.9%)	3/28 (10.7%)	1/21 (4.8%)	0.240
PVD	60/650 (9.2%)	1/17 (5.9%)	24/249 (9.6%)	23/252 (9.1%)	10/83 (12.0%)	1/28 (3.6%)	1/21 (4.8%)	0.756
COPD	70/650 (10.8%)	2/17 (11.8%)	30/249 (12.0%)	27/252 (10.7%)	5/83 (6.0%)	3/28 (10.7%)	3/21 (14.3%)	0.753
Active smoking	145/650 (22.3%)	7/17 (41.2%)	53/249 (21.3%)	58/252 (23.0%)	18/83 (21.7%)	6/28 (21.4%)	3/21 (14.3%)	0.476
CKD	310/650 (47.7%)	4/17 (23.5%)	105/249 (42.2%)	129/252 (51.2%)	43/83 (51.8%)	17/28 (60.7%)	12/21 (57.1%)	**0.043**
Preoperative dialysis	60/316 (19.4%)	1/4 (25.0%)	25/104 (24.0%)	21/126 (16.7%)	7/43 (16.3%)	4/17 (23.5%)	2/12 (16.7%)	0.394
Spondylodiscitis	45/650 (6.9%)	1/17 (5.9%)	17/249 (6.8%)	17/252 (6.7%)	6/83 (7.2%)	0/28	4/21 (19.0%)	0.226

Depending on variable distribution, metric variables are calculated as either mean, with respective standard deviation (±), or median, with [25th and 75th] percentiles. For nominal variables, the absolute number, n, is calculated with a percentage (%). Bold indicates *p* < 0.05. (%). * Fisher’s Exact test. IE: infective endocarditis; BMI: body mass index; LVEF: left ventricular ejection fraction; WBC: white blood cell count; CAD: coronary artery disease; PVD: peripheral vascular disease; COPD: chronic obstructive pulmonary disease; CKD: chronic kidney disease.

**Table 2:** Manifestations of surgically treated IE patients, according to their preoperative BMI.

Variables	All Patients n = 650	Under-Weight 17/650 (2.6%)	Normal Weight 249/650 (38.3%)	Over-Weight 252/650 (38.8%)	Obese I 83/650 (12.8%)	Obese II 28/650 (4.3%)	Obese III 21/650 (3.2%)	*p*-Value
Clinical manifestations								
Fever	373/650 (57.4%)	8/17 (47.1%)	138/249 (55.4%)	151/252 (59.9%)	51/83 (61.4%)	16/28 (57.1%)	9/21 (42.9%)	0.525
Bacteraemia	441/650 (67.8%)	13/17 (76.5%)	160/249 (64.3%)	175/252 (69.4%)	58/83 (69.9%)	19/28 (67.9%)	16/21 (76.2%)	0.673
Sepsis	259/650 (39.8%)	5/17 (29.4%)	94/249 (37.8%)	99/252 (39.3%)	35/83 (42.2%)	13/28 (46.4%)	13/21 (61.9%)	0.286
Septic embolization	215/645 (33.4%)	5/17 (29.4%)	82/247 (33.2%)	75/250 (30.0%)	34/82 (41.5%)	6/28 (21.4%)	13/21 (61.9%)	0.098
Cerebral embolization	151/650 (23.2%)	3/17 (17.6%)	57/249 (22.9%)	51/252 (20.2%)	24/83 (28.9%)	6/28 (21.4%)	10/21 (47.6%)	0.071
Neurological manifestations	180/650 (27.7%)	2/17 (11.8%)	71/249 (28.5%)	62/252 (24.6%)	28/83 (33.7)	7/28 (25.0%)	10/21 (47.6%)	0.101
Myocardial infarction	16/650 (2.5%)	0/17	6/249 (2.4%)	4/252 (1.6%)	5/83 (6.0%)	0/28	1/21 (4.8%)	0.237
Shock preoperatively	82/650 (12.9%)	1/17 (5.9%)	21/249 (8.4%)	33/252 (13.1%)	14/83 (16.9%)	6/28 (21.4%)	7/21 (33.3%)	**0.006**
Intubation preoperatively	96/650 (14.8%)	0/17	29/249 (11.6%)	34/252 (13.5%)	18/83 (21.7%)	7/28 (25.0%)	8/21 (38.1%)	**0.001**
Pacemaker IE	4/650 (0.9%)	0/17	1/249 (0.4%)	1/252 (0.4%)	0/83	1/28 (3.6%)	1/21 (4.8%)	0.053
PVE	153/637 (24.0%)	3/16 (18.8%)	56/243 (23.0%)	66/249 (26.5%)	24/80 (30.0%)	3/28 (10.7%)	1/21 (4.8%)	0.082
Echocardiographic manifestations								
Vegetations	532/641 (81.6%)	12/17 (70.6%)	201/247 (81.4%)	205/247 (83.0%)	65/82 (79.3%)	25/28 (89.3%)	16/20 (80.0%)	0.997
Perivalvular abscess	174/650 (26.7%)	3/17 (17.6%)	67/249 (26.9%)	67/252 (26.6%)	21/83 (25.3%)	9/28 (32.1%)	7/21 (33.3%)	0.890
Perforation	113/650 (17.4%)	5/17 (29.4%)	42/249 (16.9%)	48/252 (19.0%)	13/83 (15.7%)	4/28 (14.3%)	1/21 (4.8%)	0.424
Fistula	15/650 (2.3%)	1/17 (5.9%)	6/249 (2.4%)	6/252 (2.4%)	2/83 (2.4%)	0/28	0/21	0.829

For the above listed nominal variables, the absolute number, n, is calculated with a percentage (%). Bold indicates *p* < 0.05. IE: infective endocarditis; BMI: body mass index; PVE: prosthetic valve endocarditis.

**Table 3:** Operative data in surgically treated IE patients, according to their preoperative BMI.

Variables	All Patients n = 650	Under-Weight 17/650 (2.6%)	Normal Weight 249/650 (38.3%)	Over-Weight 252/650 (38.8%)	Obese I 83/650 (12.8%)	Obese II 28/650 (4.3%)	Obese III 21/650 (3.2%)	*p*-Value
Indication for surgery								
Cardiac decompensation	82/650 (12.9%)	1/17 (5.9%)	21/249 (8.4%)	33/252 (13.1%)	14/83 (16.9%)	6/28 (21.4%)	7/21 (33.3%)	**0.006**
Sepsis	259/650 (39.8%)	5/17 (29.4%)	94/249 (37.8%)	99/252 (39.3%)	35/83 (42.2%)	13/28 (46.4%)	13/21 (61.9%)	0.286
Septic embolization	215/645 (33.4%)	5/17 (29.4%)	82/247 (33.2%)	75/250 (30.0%)	34/82 (41.5%)	6/28 (21.4%)	13/21 (61.9%)	0.098
Perivalvular abscess	174/650 (26.7%)	3/17 (17.6%)	67/249 (26.9%)	67/252 (26.6%)	21/83 (25.3%)	9/28 (32.1%)	7/21 (33.3%)	0.890
Performed procedure								
Redo cardiac surgery	185/650 (27.8%)	3/17 (17.6%)	62/249 (24.9%)	81/252 (32.1%)	26/83 (31.3%)	5/28 (17.8%)	3/21 (14.3%)	0.160
Aortic valve replacement	392/650 (60.3%)	6/17 (35.3%)	142/249 (47.0%)	161/252 (63.9%)	56/83 (67.5%)	14/28 (50.0%)	13/21 (61.9%)	0.076
Mitral valve replacement	283/650 (43.5%)	11/17 (64.7%)	107/249 (42.9%)	105/252 (41.6%)	37/83 (44.6%)	13/28 (46.4%)	10/21 (47.6%)	0.309
Mitral valve repair	20/650 (3.0%)	1/17 (5.8%)	7/249 (2.8%)	7/252 (2.7%)	1/83 (1.2%)	2/28 (7.1%)	2/21 (9.5%)	0.384
Tricuspid valve replacement	27/650 (4.1%)	0/17	15/249 (6.0%)	9/252 (3.5%)	3/83 (3.6%)	0/28	0/21	0.451
Tricuspid valve repair	15/650 (2.3%)	0/17	7/249 (2.8%)	4/252 (1.6%)	2/83 (2.4%)	1/28 (3.6%)	1/21 (4.8%)	0.451
Pulmonary valve replacement	3/650 (0.5%)	0/17	2/249 (0.8%)	1/252 (0.4%)	0/83	0/28	0/21	1.000 *
Combined procedure	253/643 (39.3%)	4/17 (23.5%)	103/246 (41.9%)	98/249 (39.4%)	31/82 (37.8%)	9/28 (32.1%)	8/21 (38.1%)	0.678
Operation time (minutes)	216.2 ± 78.4	197.7 ± 78.8	210.9 ± 74.4	217.0 ± 55.2	230.8 ± 77.9	212.2 ± 82.0	248.7 ± 86.1	0.116
CPB time (minutes)	126.9 ± 58.7	110.7 ± 57.1	123.9 ± 56.7	127.0 ± 55.1	136.0 ± 57.9	118.3 ± 61.8	144.9 ± 71.3	0.163
Cross-clamp time	79.8 ± 35.3	69.5 ± 32.0	78.4 ± 36.2	79.9 ± 36.1	83.8 ± 30.4	76.9 ± 42.5	93.7 ± 41.3	0.211

Depending on variable distribution, metric variables are calculated as either mean, with respective standard deviation (±), or median, with [25th and 75th] percentiles. For nominal variables, the absolute number, n, is calculated with a percentage (%).* Fisher’s Exact test; IE: infective endocarditis; BMI: body mass index; CPB: cardiopulmonary bypass.

**Table 4:** Outcomes according to preoperative BMI in surgically treated IE patients.

Variables	All Patients n = 650	Under-Weight 17/650 (2.6%)	Normal Weight 249/650 (38.3%)	Over-Weight 252/650 (38.8%)	Obese I 83/650 (12.8%)	Obese II 28/650 (4.3%)	Obese III 21/650 (3.2%)	*p*-Value
Re-thoracotomy	97/650 (15.0%)	1/17 (5.9%)	33/249 (13.3%)	44/252 (17.5%)	12/83 (14.5%)	5/28 (17.9%)	2/21 (9.5%)	0.746
New pacemaker implantation	69/648 (10.6%)	1/17 (5.9%)	22/249 (8.8%)	29/251 (11.6%)	9/82 (11.0%)	5/28 (17.9%)	3/21 (14.3%)	0.205
Myocardial infarction	6/647 (0.9%)	0/17	2/248 (0.8%)	3/251 (1.2%)	1/82 (1.2%)	0/28	0/21	0.968
CVE	31/647 (4.8%)	1/17 (5.9%)	11/248 (4.4%)	15/250 (6.0%)	4/83 (4.8%)	0/28	0/21	0.642
AKI	203/648 (31.3%)	3/17 (17.6%)	63/249 (25.3%)	90/251 (35.9%)	24/83 (28.9%)	12/27 (44.4%)	11/21 (52.4%)	**0.011**
Dialysis postoperatively	91/200 (45.5%)	0/3	24/63 (38.1%)	39/87 (44.8%)	13/24 (54.2%)	9/12 (75.0%)	6/11 (54.5%)	0.105
Sternal wound infection	20/441 (4.5%)	1/13 (7.7%)	7/164 (4.3%)	7/173 (4.0%)	1/60 (1.7%))	1/17 (5.9%)	3/14 (21.4%)	**0.002**
Septic shock	37/104 (35.6%)	1/3 (33.3%)	11/40 (27.5%)	11/37 (29.7%)	3/9 (33.3%)	5/6 (83.3%)	5/7 (71.4%)	0.354
Intubation time (hours)	24.0 [10.6–144.0]	84.7 ± 233.9	235.2 ± 884.9	151.0 ± 345.1	106.9 ± 282.9	179.9 ± 191.2	104.0 ± 101.0	0.357
Tracheotomy	87/647 (13.5%)	1/17 (5.9%)	27/249 (10.8%)	41/250 (16.4%)	8/82 (9.8%)	6/28 (21.4%)	4/21 (19.0%)	0.205
ICU stay duration (days)	5.0 ± 6.3	5.0 ± 5.8	7.3 ± 8.3	8.1 ± 9.5	7.6 ± 9.4	8.9 ± 6.4	7.2 ± 4.6	0.403
In-hospital stay (days)	13 [9.7–19.0]	13 [9.3–18.0]	13 [9.0–18.0]	13 [10.0–21.0]	13 [10.0–18.0]	13 [7.0–15.0]	13 [7.0–13.0]	0.061
30-day mortality	85/648 (13.1%)	2/17 (11.8%)	23/248 (9.3%)	36/251 (14.3%)	14/83 (16.9%)	6/28 (36.4%)	4/21 (19.0%)	0.225
1-year mortality	162/501 (32.3%)	5/13 (38.5%)	55/179 (30.7%	62/203 (30.5%)	21/66 (31.8%)	9/22 (40.9%)	10/18 (55.6%)	0.314
Readmission	205/402 (51.0%)	7/11 (63.6%)	73/153 (47.7%)	83/158 (52.5%)	25/51 (49.0%)	8/15 (53.3%)	9/14 (64.3%)	0.759
IE recurrence	25/398 (6.3%)	1/11 (9.1%)	13/152 (8.6%)	7/155 (4.5%)	3/51 (5.9%)	1/15 (6.7%)	0/14	0.661

Depending on variable distribution, metric variables are calculated as either mean, with respective standard deviation (±), or median, with [25th and 75th] percentiles. For nominal variables, the absolute number, n, is calculated with a percentage (%). Each *p*-value refers to the comparison of the variable in the preceding BMI category to the remaining series. Bold indicates *p* < 0.05. IE: infective endocarditis; BMI: body mass index; ICU: intensive care unit; CVE: cerebrovascular events; AKI: acute kidney injury.

**Table 5:** Risk estimates for 30-day and 1-year mortality, according to BMI (kg/m^2^).

30-Day Mortality			1-Year Mortality		
	Odds Ratio [95%–CI]	*p*-Value		Hazard Ratio [95%–CI]	*p*-Value
Univariable					
BMI ≥ 30	1.430 [0.860–2.378]	0.167	BMI ≥ 30	1.312 [0.840–2.047]	0.232
BMI ≥ 35	2.377 [1.192–4.739]	0.012 *	BMI ≥ 35	1.955 [1.020–3.746]	0.040 *
BMI ≥ 40	2.095 [0.767–5.724]	0.141	BMI ≥ 40	2.650 [1.026–6.844]	0.037 *
Multivariable					
BMI ≥ 35	1.257 [0.613–2.579]	0.533	BMI ≥ 35	1.157 [0.564–2.374]	0.690
			BMI ≥ 40	1.487 [0.448–2.304]	0.969

* statistically significant.

**Table 6:** Independent risk factors for 30-day and 1-year mortality after a multivariable regression.

30-Day Mortality			1-Year Mortality		
Preoperative Risk Factor	OR [95%–CI]	*p*-Value	Preoperative Risk Factor	HR [95%–CI]	*p*-Value
Age > 60 years	2.415 [1.425–4.091]	**0.001**	Age > 60 years	2.878 [1.809–4.579]	**<0.001**
Staphylococcus aureus	2.122 [1.308–3.443]	**0.002**	Staphylococcus aureus	2.035 [1.305–3.172]	**0.002**
LVEF < 30%	3.801 [1.287–11.227]	**0.016**	PVE	1.983 [1.279–3.075]	**0.002**

Bold indicates *p* < 0.05; OR: odds ratio; CI: confidence interval; HR: hazard ratio; LVEF: left ventricular ejection fraction; PVE: prosthetic valve endocarditis. Graphical abstract.

## Data Availability

The data that support the findings of this study are available from the corresponding author (A.E) upon reasonable request.

## Supplementary Materials

The following supporting information can be downloaded at: https://www.mdpi.com/article/10.3390/jcdd11100327/s1, Table S1: Distribution of risk factors in surgically treated IE patients by preoperative BMI; Table S2: Microbiological data in surgically treated IE patients by preoperative BMI; Table S3: A subanalysis of outcomes by BMI with combined groups; Figure S1: Scatterplot of patient BMI versus survival time.
