# Supplementary material for: Body Weight’s Role in Infective Endocarditis Surgery
Source: J Cardiovasc Dev Dis. 2024 Oct 15;11(10):327. doi: 10.3390/jcdd11100327 (PMC11508204; doi:10.3390/jcdd11100327)
Supplement: Supplementary file 1 [file jcdd-11-00327-s001.zip › jcdd-3180955-supplementary.pdf]

Body Weight's Role in Infective Endocarditis Surgery

Supplementary Digital Material

Supplementary Table S1: Distribution of risk factors in surgically treated IE patients by preoperative BMI

Variables	**All patients** n=650	**Under-weight** 17/650 (2.6%)	**Normal weight** 249/650 (38.3%)	**Over-weight** 252/650 (38.8%)	**Obese I** 83/650 (12.8%)	**Obese II** 28/650 (4.3%)	**Obese III** 21/650 (3.2%)	*p*-value
Underlying Valve lesions	199/650 (30.6%)	4/17 (23.5%)	74/249 (29.7%)	85/252 (33.7%)	26/83 (31.3%)	6/28 (21.4%)	4/21 (19.0%)	0.535
Mitral valve prolapse	31/650 (4.7%)	5/17 (29.4%)	15/249 (6.0%)	5/252 (2.0%)	6/83 (7.2%)	0/28	0/21	**<0.001**
Congenital heart disease	18/650 (2.8%)	0/17	9/249 (3.6%)	7/252 (2.8%)	1/83 (1.2%)	0/28	1/21 (4.8%)	0.699
Valve prosthesis	154/650 (23.6%)	3/17 (17.6%)	55/249 (22.1%)	67/252 (26.6%)	24/83 (28.9%)	4/28 (14.3%)	1/21 (4.8%)	0.124
Vascular prosthesis	18/650 (2.7%)	0/17	3/249 (1.2%)	10/252 (4.0%)	5/83 (6.0%)	0/28	0/21	0.119
Previous Pacemaker	51/645 (7.9%)	2/16 (12.5%)	13/247 (5.3%)	25/252 (10.1%)	4/81 (4.9%)	4/28 (14.3%)	3/21 (14.3%)	0.150
IE history	45/650 (6.9%)	3/17 (17.6%)	19/249 (7.6%)	13/252 (5.2%)	6/83 (7.2%)	4/28 (14.3%)	0/21	0.137
Active Malignancy	55/650	2/17	27/249	19/252	5/83	2/28	0/21	0.418

	(8.4%)	(11.8%)	(10.8%)	(7.5%)	(6.0%)	(7.1%)		
Alcohol dependency	51/650 (7.8%)	2/17 (11.8%)	17/249 (6.8%)	21/252 (8.3%)	7/83 (8.4%)	3/28 (10.7%)	1/21 (4.8%)	0.920
Intravenous Drug abuse	43/650 (6.6%)	2/17 (11.8%)	29/249 (11.6%)	10/252 (4.0%)	1/83 (1.2%)	1/28 (3.6%)	0/21	**0.001**

For each of the nominal variables listed, the absolute number, n, was calculated with a percentage (%). **Bold** indicates $p<0.05$.

IE: Infective Endocarditis, BMI: Body Mass Index.

Supplementary Table S2: Microbiological data in surgically treated IE patients by preoperative BMI

Variables	**All patients** n=650	**Under-weight** 17/650 (2.6%)	**Normal weight** 249/650 (38.3%)	**Over-weight** 252/650 (38.8%)	**Obese I** 83/650 (12.8%)	**Obese II** 28/650 (4.3%)	**Obese III** 21/650 (3.2%)	*p*-value
Known organism	589/649 (90.8%)	16/17 (94.1%)	223/249 (89.6%)	232/251 (92.4%)	71/83 (85.5%)	28/28 (100%)	19/21 (90.5%)	0.218
Positive blood culture	527/588 (89.6%)	15/16 (93.8%)	201/223 (90.1%)	204/231 (88.3%)	65/71 (91.5%)	24/28 (85.7%)	18/19 (94.7%)	0.841
Positive valve microbiology	208/581 (35.8%)	6/16 (37.5%)	71/222 (32.0%)	90/228 (39.5%)	22/69 (31.9%)	11/27 (40.7%)	8/19 (42.1%)	0.572
Staphylococcus aureus	171/650 (26.3%)	3/17 (17.6%)	60/249 (24.1%)	63/252 (25.0%)	24/83 (28.9%)	13/28 (46.4%)	8/21 (38.1%)	0.103
CoNS	90/650 (13.8%)	2/17 (11.8%)	32/2249 (12.9%)	44/252 (17.5%)	9/83 (10.8%)	2/28 (7.1%)	1/21 (4.8%)	0.291
Streptococci	173/650 (26.6%)	3/17 (17.6%)	71/249 (28.5%)	66/252 (26.2%)	21/83 (25.3%)	8/28 (28.6%)	4/21 (19.0%)	0.859
Enterococci	94/650 (14.5%)	5/17 (29.4%)	30/249 (12.0%)	40/252 (15.9%)	11/83 (13.3%)	2/28 (7.1%)	6/21 (28.6%)	0.096

Gram-negative HACEK	5/650 (0.8%)	0/17	4/249 (1.6%)	1/252 (0.4%)	0/83	0/28	0/21	0.564
Gram-negative non-HACEK	23/650 (3.5%)	2/17 (11.8%)	10/249 (4.0%)	6/252 (2.4%)	2/83 (2.4%)	3/28 (10.7%)	0/21	0.080
Fungi	8/650 (1.2%)	0/17	3/249 (1.2%)	3/252 (1.2%)	0/83	1/28 (3.6%)	1/21 (4.8%)	0.458
Other Organisms	51/650 (7.8%)	2/17 (11.8%)	21/249 (8.4%)	19/252 (7.5%)	5/83 (6.0%)	3/28 (10.7%)	1/21 (4.8%)	0.914

For listed nominal variables, the absolute number, n, is calculated with percentage (%). **Bold** indicates $p<0.05$.

IE: infective endocarditis. CoNS: coagulase-negative staphylococci. HACEK: Haemophilus species, Aggregatibacter species, Cardiobacterium hominis, Eikenella corrodens and Kingella species.

Supplementary Table S3: A subanalysis of outcomes by BMI with combined groups.

Variables	**BMI<25** 283/650 (43.5%)	**BMI 25-35** 335/650 (51.5%)	**BMI>35** 49/650 (7.5%)	*p*-value
Re-thoracotomy	36/283 (12.7%)	56/335 (16.7%)	7/49 (14.3%)	0.374
Tracheotomy	27/249 (10.8%)	41/250 (16.4%)	8/82 (9.8%)	0.160
New pacemaker implantation	24/277 (8.7%)	38/333 (11.4%)	8/49 (16.3%)	0.221
Myocardial infarction	2/276 (0.7%)	4/333 (1.2%)	0/49	0.648
CVE	12/276 (4.3%)	19/333 (5.7%)	0/49	0.198
AKI	71/277 (25.6%)	114/334 (34.1%)	23/48 (47.9%)	**0.003**
Sternal wound infection	8/177 (4.5%)	8/233 (3.4%)	4/31 (12.9%)	**<0.001**

30-day mortality	25/265 (9.4%)	50/334 (15.0%)	10/49 (20.4%)	**0.031**
1-year mortality	66/202 (32.7%)	83/269 (30.9%)	19/40 (47.5%)	0.112

For above listed nominal variables, the absolute number, n, is calculated with percentage (%). **Bold** indicates $p<0.05$. IE: infective endocarditis, BMI: Body mass index, CVE: Cerebrovascular Events, AKI: Acute Kidney Injury.

Supplementary Figure S1: Scatterplot of patient BMI versus survival time.

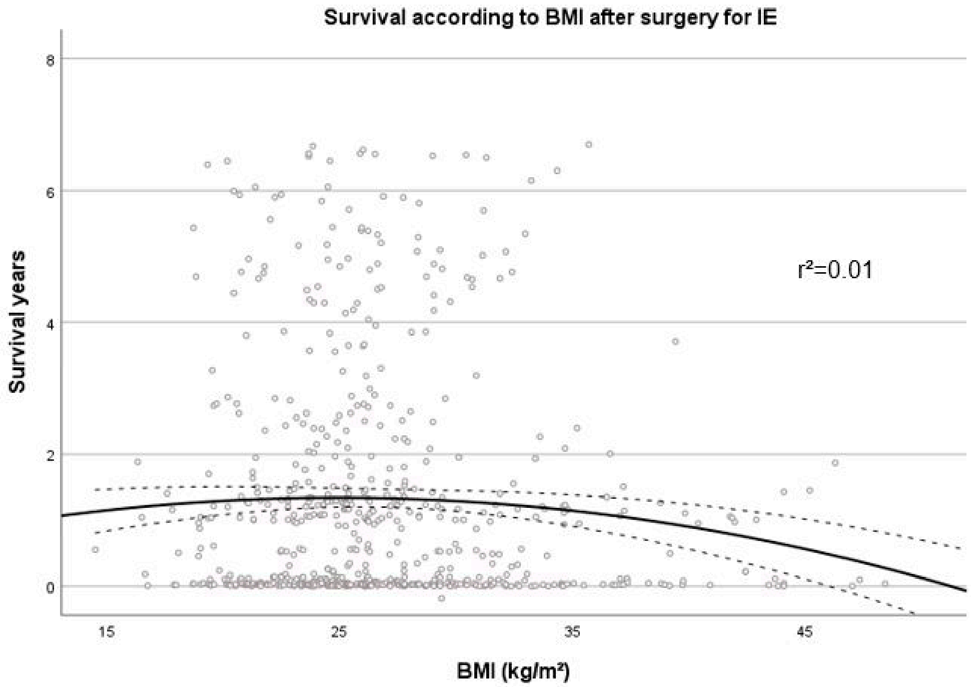

Scatterplot of patient BMI versus survival time.

Each dot represents a unique case. The solid line represents the median regression, and the dotted lines the 95% confidence intervals.
